# Spinal manipulation and modulation of pain sensitivity in persistent low back pain: a secondary cluster analysis of a randomized trial

**DOI:** 10.1186/s12998-021-00367-4

**Published:** 2021-02-24

**Authors:** Casper Glissmann Nim, Kenneth Arnold Weber, Gregory Neill Kawchuk, Søren O’Neill

**Affiliations:** 1 Medical Research Unit, Spine Center of Southern Denmark, University Hospital of Southern Denmark, Østrehougvej 55, 5500 Middelfart, Denmark; 2 Department of Regional Health Research, University of Southern Denmark, Campusvej 55, 5230 Odense, Denmark; 3 Department of Anesthesiology, Perioperative and Pain Medicine, Stanford University, Stanford, USA; 4 Department of Physical Therapy, University of Alberta, Edmonton, Canada

**Keywords:** Low back pain, Chronic pain, Spinal manipulation, Quantitative sensory testing, Pain modulation, Pressure pain threshold

## Abstract

**Background:**

Pain hypersensitivity can be assessed using Quantitative Sensory Testing (QST) and is associated with persistent low back pain. Spinal manipulation appears to modify pain hypersensitivity, and this could function as one mechanism leading to clinical improvements. In the current study, we applied a comprehensive QST battery to assess pain sensitivity in a cohort of low back pain patients before and after spinal manipulation to improve our understanding of the association between QST and clinical improvements. This study addresses two questions: Are clinical improvements following spinal manipulation in low back pain patients contingent on pain hypersensitivity, and does pain sensitivity change following spinal manipulation?

**Methods:**

We performed a secondary analysis of data from a randomized clinical trial. One hundred and thirty-two participants with persistent LBP were treated with spinal manipulation four times over two weeks. Patient-reported outcomes and QST were assessed at baseline, after the fourth spinal manipulation session, and 14-days later. The clinical outcomes were changes in low back pain intensity and disability. Using latent profile analysis, we categorized the participants into clusters depending on their baseline QST scores. We used linear mixed models to examine the association between clusters and changes in patient-reported outcomes and QST.

**Results:**

Two clusters emerged: a *Sensitized* and a *Not sensitized*. The former had significantly lower regional pressure and thermal pain thresholds, remote pressure pain tolerance, and lower inhibitory conditioned pain modulation than the Not sensitized group. However, we only found between-cluster differences for regional pressure pain threshold following spinal manipulation. Thus, the clusters were not associated with patient-reported pain and disability changes or the remaining QST outcomes.

**Conclusions:**

We report that the baseline QST profile was not associated with clinical improvements following spinal manipulation. We did observe a substantial change for regional pressure pain threshold, which suggests that any effect of spinal manipulation on pain sensitivity is most likely to be observed as changes in regional, mechanical pain threshold. However, the mechanism that invokes clinical improvement and pain sensitivity changes appear distinct. Due to methodological caveats, we advise caution when interpreting the results.

**Trial registration:**

Clinical.Trial.gov identifier: NCT04086667, registered 11 September 2019 – Retrospectively registered, https://clinicaltrials.gov/ct2/show/NCT04086667

**Supplementary Information:**

The online version contains supplementary material available at 10.1186/s12998-021-00367-4.

## Background

The conscious experience of pain is not a simple reflection of the stimulus, which caused it. A stimulus may initiate the conduction of a nociceptive signal, but the signal can be heavily modulated in the nervous system before reaching consciousness as pain. Long-lasting pain can cause disturbances in this system, resulting in pain hypersensitivity [1]. Pain sensitivity and pain modulation can be examined using standardized psychophysical measures, collectively known as Quantitative Sensory Testing (QST) [2]. Hence, we are able to quantify an individual’s pain sensitivity using their pain perception elicited by a standardized stimulus [3]. This allows us to describe different types of pain and treatment-induced changes within the same pain cohort.

Persistent low-back pain (LBP) patients appear to be affected by pain hypersensitivity and perturbations in pain modulation. When comparing persistent LBP patients to healthy individuals using QST, large differences are noted [4]. While this arguably constitutes one process in developing persistent pain [5], the predictive value of baseline QST is unfortunately questionable and not sufficiently researched [6–8]. Nonetheless, studies have reported QST changes following successful treatment in experimental [9, 10] and clinical settings [11], arguably reflecting an underlying mechanistic explanation of pain relief.

Spinal manipulation (SM) is often used to treat low back pain and is historically theorized to effect clinical changes through neurophysiological mechanisms [12]. The study of changes in pain sensitivity following SM has received considerable attention in the field. No less than four systematic reviews are available on the subject [13–16], and while there is an apparent disparity in the research, it appears that SM affects regional mechanical pain sensitivity across pain populations. To investigate this further, we recently published a randomized trial investigating whether the SM’s application site (a stiff vs. a pain-sensitive segment) moderated the observed effect on regional pressure pain thresholds [17], and that study indicated a segmental effect. Namely, that regional pressure pain threshold increased for the group receiving SM at a pain-sensitive segment compared to the group receiving SM at a segment characterized by stiffness. We postulated that this was purely a segmental neurophysiological reflex phenomenon, as this effect was disconnected from a clinical pain reduction.

### Objectives

Our previous study questioned the clinical benefit of increases in pressure pain thresholds. Furthermore, the findings have generated multiple questions concerning the association between baseline pain hypersensitivity and changes in i) patient-reported pain and disability and ii) QST outcomes following SM. Hence, this secondary analysis will re-analyze the longitudinal data used in the original randomized clinical trial [17] to answer the following specific research aims:

1. To cluster patients with persistent LBP cross-sectionally using a data-driven approach based on the baseline QST data.
2. To determine whether short-term changes in patient-reported pain and disability and QST outcomes differ between clusters.

## Method

### Setting

A secondary analysis using data from a randomized clinical trial (Clinical.Trial.gov identifier: NCT04086667, registered 11 September 2019 – Retrospectively registered, https://clinicaltrials.gov/ct2/show/NCT04086667) [17]. The sample consisted of 132 participants with non-specific persistent LBP. All participants were recruited from a secondary-care hospital outpatient Spine Center in Southern Denmark between November 2017 and February 2019. The full description of participants, eligibility criteria and study procedures are reported elsewhere [17]. Here we provide a summary of the methods pertinent to the current analysis.

Inclusion criteria of the study included: benign and non-specific LBP (not malignant, infectious, inflammatory, or fracture) for more than three months, no prior spinal surgery, no current surgical indications due to radiculopathy, and age between 18 and 60 years. All participants gave oral and written informed consent for the study approved by the regional research ethics board (S-20160201).

### Spinal manipulation

The SM was provided in a standardized manner with the participant in a side-lying position. A high velocity, low amplitude thrust targeted the randomized segment (the stiffest or the most pain-sensitive) in a posterior to anterior direction. The contact point was at the spinous process. We allowed up to three SM attempts for a successful treatment. The chiropractor who provided the SM determined subjectively and independent of cavitation whether the treatment was successful. Two chiropractors performed all SMT in this study, each with more than 12 years of clinical experience. A total of four SM sessions were provided over 14 days [17].

### Procedure

The study design included three visits to the Human Experimental Pain Laboratory at the Spine Center of Southern Denmark:

- Before the first SM treatment session (baseline)
- Immediately following the fourth and final SM treatment (post-SM)
- Two weeks after the end of SM treatment (follow-up)

We collected data on patient-reported pain and disability and QST at each of the laboratory visits. After enrollment, the participants were randomized to receive SM directed at either the segment of highest stiffness, measured using the VerteTrack [18], or the segment of lowest mechanical pain threshold measured using pressure algometry [19].

### Data collection

#### Patient-reported outcomes

The Low Back Pain Rating Scale was used to assess low back pain intensity. Three 11-point numerical rating scales (NRS) quantified the current, worst, and average LBP over the last 14 days. These scores were combined into a mean score [0–10], with 0 indicating no and 10 indicating the worst imaginable pain. The scale is reliable for assessing LBP intensity [20].

The Oswestry Disability Index (ODI) version 2.1 [21] was used to measure disability. The ODI is a 10-item questionnaire with a five-point Likert rating scale, ranging from no disability to high disability. The items were combined and converted into percentages [0–100%]. This outcome measure has been translated into Danish and is responsive to clinical changes [22].

#### Quantitative sensory testing outcomes

The QST consisted of six different tests using three different methods. We measured pain sensitivity regionally (at the lower back) and remote (at the legs). The definition of the different QST procedures can be found in Table 1 [3, 23, 24]:

**Table 1:** Definitions of the different QST procedures

QST procedure	Definition
*Pain threshold*	The minimum intensity of a stimulus that is perceived as painful
*Pain tolerance*	The maximum intensity of a pain-producing stimulus tolerated
*Temporal summation*	Increases in subjective pain response during repetitive pain-producing stimuli
*Conditioned pain modulation*	Decreases (inhibitory) or increases (facilitatory) of the pain response during the application of pain-producing conditioning stimulus

*QST:*  Quantitative sensory testing

#### Static regional quantitative sensory testing

Manual pressure algometry (model 2, Somedic, Sweden) was used to assess regional pressure pain threshold at the lower back. With the participant placed in the prone position, we gradually increased the pressure perpendicular to the skin, simultaneously on both sides of each spinous process from L1 to L5 at a rate of 50 kPa/s using a custom made double-headed probe (Supplementary file 1). The participant indicated the onset of pain by pressing a button, which discontinued the pressure and recorded the pressure pain threshold. Each of the five segments was tested three times in random order. If no pain was elicited at 1000 kPa, we recorded 1000 kPA as the regional pressure pain threshold. Pressure algometry has excellent intra-rater reliability in LBP patients [25].

A thermode (Medoc TSA-II, Israel) with a single 3 cm × 3 cm probe was applied at the midline for each spine process (L1 to L5) to measure regional heat pain threshold. The thermode baseline temperature was pre-set to 32 °C. During testing, it increased at a rate of 1 °C/s until the participants indicated the stimulation as painful by pressing an indicator button. When indicated as painful, the probe was lifted off the skin, and the temperature returned to the baseline temperature (10 °C/s). If no pain was elicited at 50 °C, 50 °C was recorded as the regional heat pain threshold, and the thermode returned automatically to the baseline temperature. Regional heat pain threshold measured at the spine has good-to-excellent intra-rater reliability in a healthy population [26].

For both regional pressure pain threshold and regional heat pain threshold, we first completed a trial procedure consisting of 1–2 tests on the lower extremity and at the T12 spinal segment to familiarize the participant with the procedure before testing. We used a composite score for each of the regional pressure pain threshold and regional heat pain threshold. The three tests at each segment were averaged into a single segment score, then the average between all segments was calculated and used for the analysis [17].

#### Remote quantitative sensory testing

Computer-controlled cuff algometry (CCA) on the lower extremities was used to measure static remote pressure pain threshold and pressure pain tolerance threshold, and dynamic temporal summation, and conditioned pain modulation. The CCA procedure employed two 13-cm wide silicone tourniquet cuffs (VBM, Sulz, Germany). Each with two adjacent, equally sized proximal and distal chambers wrapped around the non-dominant and dominant gastrocnemius muscle 5 cm inferior to the tibial tuberosity. The pain intensity was assessed by increasing the inflation of the cuff.

#### Remote static quantitative sensory testing

We used the dominant leg as the experimental test site, which assessed deep-tissue pain sensitivity as a stimulus-response curve. The cuff pressure increased with 1 kPa/s in both chambers; the pressure limit was 100 kPa. Participants indicated their pain on a computerized electronic visual analog scale (VAS) (“No pain” = 0 cm to “Worst pain imaginable” = 10 cm). We instructed the participants to continuously rate the induced pressure pain intensity from the initial pain onset. The pressure at which the participant first perceived the stimulus as painful was noted as remote pressure pain threshold. The pressure at the time of termination was recorded as remote pressure pain tolerance threshold. If cuff pressure was tolerated to the limit of 100 kPa, 100 kPA was recorded as the remote pressure pain tolerance threshold, and the pressure was instantly released.

#### Remote dynamic quantitative sensory testing

The CCA was programmed to apply a series of 10 pulses to the dominant leg of equal pressure to the individual’s remote pressure pain tolerance threshold at a rate of 1 Hz (i.e., 1 s of inflation to the target pressure and 1 s of deflation). The average pressure pain intensity of the first three stimuli was subtracted from the average of the last three stimuli and recorded as the temporal summation. We recorded Conditioned Pain Modulation as the difference in remote pressure pain threshold at the dominant leg (test stimulus) before and during continuous conditioning pressure stimulus applied to the non-dominant leg (conditioning stimulus at 70% of the individual remote pressure pain tolerance threshold). The CCA has previously been used to quantify temporal summation and conditioned pain modulation and is deemed reliable and sensitive for changes [27].

### Statistical analysis

#### Latent profile analysis

We used a data-driven latent profile analysis to develop a clustering model with baseline QST variables for the participants with complete QST datasets (regional pressure pain threshold, regional heat pain threshold, remote pressure pain threshold, remote pressure pain tolerance threshold, temporal summation, and conditioned pain modulation). No generally accepted strategy exists to determine the sample size for such an analysis. However, 2^k^ participants have been suggested to be sufficient, where *k* denotes the number of variables included in the model [28]. To assess the independence of the QST variables, we calculated correlation coefficients between each variable. A priori, we decided that if two variables were strongly correlated (coefficient > 0.7), one of the variables would be omitted from the model [29]. As the six variables were quantified using different continuous scales and limits, the variables were Z-transformed (mean-centered and normalized to one standard deviation). We reversed the temporal summation score to ease interpretation, as the meaning is the opposite of the remaining tests (a higher score indicating *higher pain sensitivity*).

We fitted the clustering models using the *Mclust package* for R [30]. In addition to the number of clusters, *Mclust* uses different covariance structures to make the model as parsimonious as possible. A minimum of 2 clusters and a maximum of 6 clusters were investigated. We used the Bayesian Information Criterion (BIC) to evaluate the number of clusters, where the highest negative number indicates the best model fit [31]. Another component cluster was only added if the BIC score improved by two units [32]. Afterward, we performed the Bootstrap Likelihood Ratio Test, which compares model fit between different numbers of clusters, i.e., whether an increase in clusters increases fit [33]. Finally, we present the fitted model using the following parameters: number of clusters, data structure, BIC, probability of belonging to a particular cluster, the results from the Bootstrap Likelihood Ratio Test, and individuals in each cluster. Any unexpected result of the latent profile analysis would be examined in a posthoc test.

We present the scaled means of each QST parameter per cluster. To describe the stability of the QST scores in groups at the different time points, the baseline fitted model was applied cross-sectionally to the additional time points (post-SM and follow-up) and presented as a horizontal process flowchart.

#### Baseline differences between clusters

We present baseline differences between the clusters as medians with interquartile ranges or count and frequency. We compare the groups using appropriate univariate testing corresponding to the data type. We compared the clusters on the patient-reported measures, the QSTs, and the participants’ sex, age, and psychological profile using three short-item variables i) depression, ii) catastrophization, and iii) anxiety - all variables associated with a persistent back pain outcome [34, 35]. The demographic and psychological data were obtained from the SpineData questionnaire [36]. The psychological constraints were one or two short item questions ranging from 0 to 10, where 0 indicates no psychological affection, and 10 indicates high psychological affection. The Concurrent validity for these short item question is comparable to the original questionnaires when tested in a similar setting [37].

#### Outcomes following spinal manipulation

We constructed linear mixed models to investigate changes in patient-reported pain, disability (NRS and ODI), and the QSTs following SM. The model assumptions were normal distribution of the residuals errors and homogeneity of the variance, which were assessed using QQ-plots and plotting the residuals versus the predicted values. We applied the final cluster model and time as interacting fixed effects, with the participant as the random intercept. We present the within-cluster mean changes at post-SM and follow-up compared to baseline and the resulting between-cluster mean differences for each model with 95% confidence intervals.

While we omitted the randomization process, we did previously observe changes in regional pressure pain threshold modified by the SM allocation site [17]. Therefore, a posthoc test was planned should the clustering result in significant interactions for the QST outcomes. This posthoc test would apply a three-way analysis approach (cluster:time:SM allocation site). Again, we present the within-cluster mean changes and between-cluster mean differences with 95% confidence intervals.

We completed data cleaning and analyses in R [38] (Linux, v. 3.6 with R-studio v. 1.3), using the *Tidyverse* [39], 95% confidence intervals and *p*-values for the mixed models were calculated using the *LMERtest* package [40]. A *p*-value of less than 0.05 indicated statistical significance for all statistical tests.

## Results

### Latent profile analysis

Datasets from 5 participants were incomplete, consequently, the sample size used for the analysis was 127. We found no strong correlations between any of the QST variables. Therefore, we included all six QST variables in the models (see Supplementary file 2). With six QST variables, a minimum of 64 [26] samples would be necessary, indicating that our sample size was sufficient for the model clustering.

Table 2 shows the top three BIC models. The model 2-VEE (rank #2) was the second most optimal model, but the difference in BIC score from the 3-VII model (rank #1) was − 0.40, i.e., less than 2.00 [32]. The Likelihood Ratio Test demonstrated a significantly better fit between 1 and 2 groups, but not between 2 and 3 (*p*-value of 0.11). Therefore, we chose the 2-VEE solution as the optimal and most parsimonious model. The 2-VEE data structure indicated the following: i) 2 clusters, ii) the distribution is ellipsoidal, iii) the volume is variable, iv) the shape is equal, and v) the orientation is equal [30].

**Table 2:** The top 3 models with the highest BIC score used to measure the fit of quantitative sensory tests derived by latent profile analysis

Rank	Data structure	n clusters	BIC score	BIC Difference
*1*	VII	3	− 2152	0
*2*	VEE	2	−2152	−0.4
*3*	VII	2	− 2155	−2.6

For a detailed overview of the VEE,2 model, see Table 3. An acceptable rate of 85% of the participants had a probability of more than 90% of belonging to the specific cluster [41].

**Table 3:** Latent profile analysis measures of model fit and cluster probability for the chosen model, 2-VEE

BIC	Probability (> 90%)	The Likelihood Ratio Test (p-value)	n cluster
*− 2152*	0.85	2 vs 3 groups: 22.2 (0.11)	1:38/2:89

**Fig. 1:**
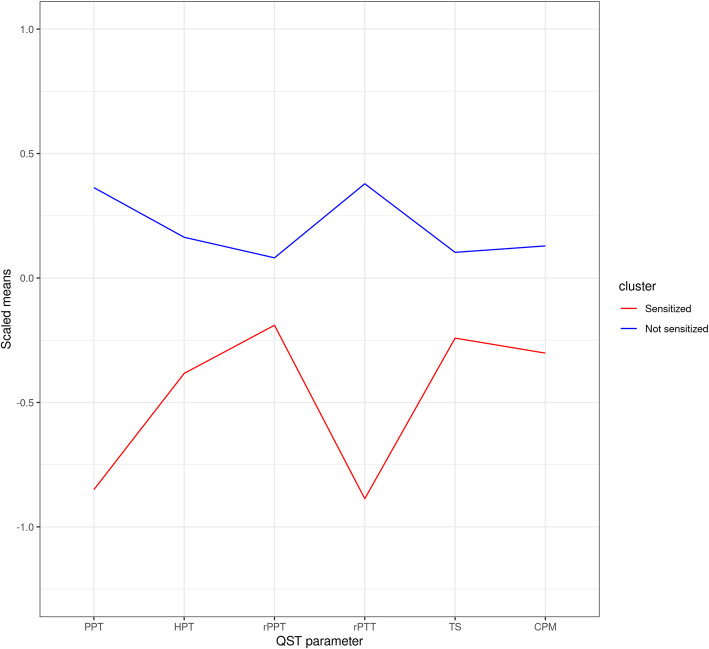
Quantitative sensory tests for the two groups derived from latent profile analysis. Scaled mean values for the different QST parameters [mean = 0, standard deviation = 1]. QST: Quantitative sensory pain test. PPT: Regional pressure pain threshold. HPT: Regional heat pain threshold, rPPT: Remote pressure pain threshold, rPTT: Remote pressure pain tolerance threshold, TS: Temporal summation, CPM: Conditioned pain modulation

Based on the profile of the QST measures of each cluster, we designated the clusters as *Sensitized* and *Not sensitized* groups. The Sensitized group had lower regional pressure pain thresholds, regional heat pain thresholds, remote pressure pain thresholds, and tolerances. They also had lower inhibitory conditioned pain modulation scores, indicating more pain hypersensitivity than the Not sensitized group. Temporal summation was scored opposite to our expectation, e.g., the Not sensitized group had a higher temporal summation score indicating higher pain hypersensitivity. See Fig. 1 for a visual illustration of the distribution between clusters.

**Fig. 2:**
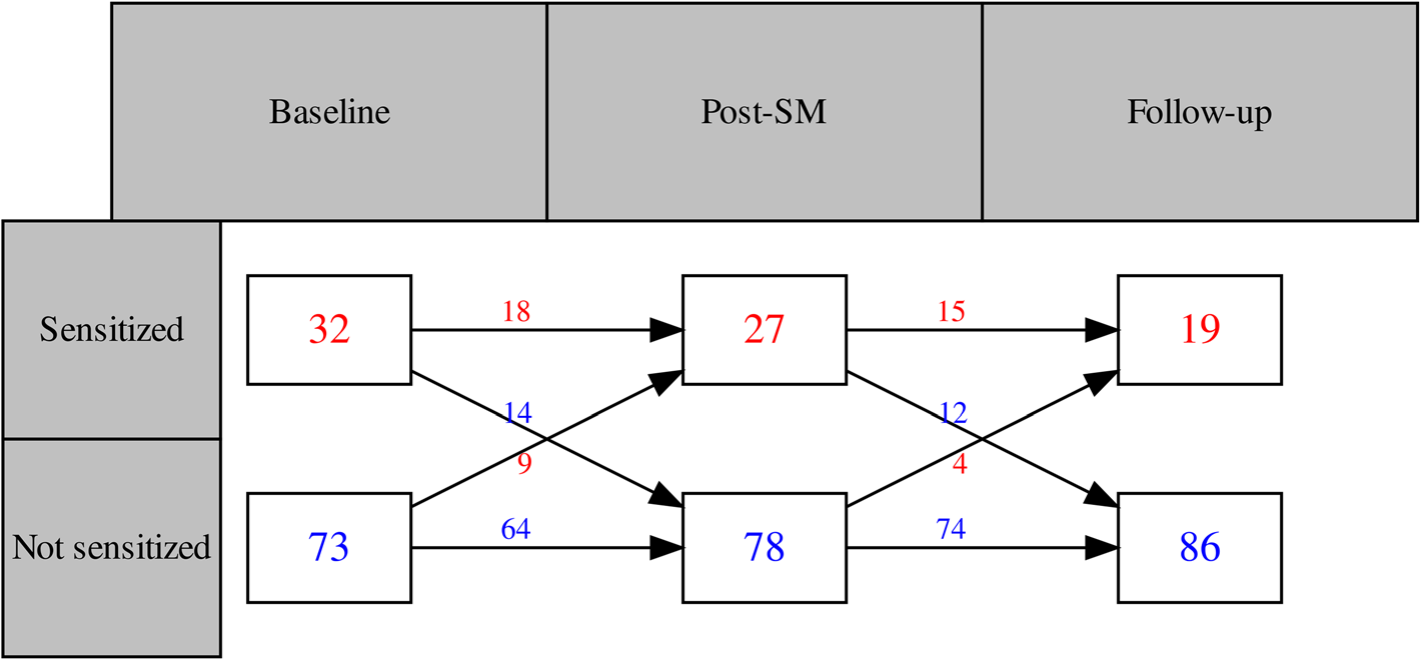
Stability patterns of quantitative sensory tests for two groups at three different time points. An overview of the cluster stability pattern in the latent profile analysis following spinal manipulation in persistent low back pain patients. The numbers above the arrows indicate the transition of patients over time. A red number indicates the transition to Sensitized, and a blue number indicates the transition to Not sensitized

Of the participants with complete QST data at follow-up (*n* = 105), 73 were Not sensitized at baseline, and 32 were noted as Sensitized. However, at follow-up, the number of Sensitized participants dropped to 19, as indicated by increases in the scaled QST scores (approximating 1). Figure 2 illustrates this relation.

### Baseline differences between clusters

Table 4 lists the baseline differences between the two clusters. The clusters differ when applying univariate testing for: regional pressure and thermal pain thresholds, remote pressure pain tolerance, and conditioned pain modulation. Noticeably, the clusters did not differ for any patient-reported outcome, nor on any of the psychological variables.

**Table 4:** Baseline differences between each latent profile derived cluster using QST in chronic low back pain

Variable	Sensitized	Not sensitized	p-value
*Low back pain intensity*	6.17 (5.00, 7.25)	5.33 (4.33, 6.67)	0.07
*Oswestry disability index*	30 (20, 40)	26 (16, 34)	0.1
*sex*			0.1
*Female*	22 (58%)	36 (40%)	
*Male*	16 (42%)	53 (60%)	
*Age*	44 (39, 50)	47 (39, 54)	0.2
*Depression*	3.75 (2.12, 5.50)	4.00 (1.00, 6.00)	0.8
*Catastrophization*	4.00 (2.00, 6.00)	3.75 (2.00, 5.50)	0.6
*Anxiety*	5 (1, 7)	4 (2, 5)	0.3
*Regional pressure pain threshold*	266 (194, 387)	586 (399, 697)	< 0.001
*Regional heat pain threshold*	40.6 (38.9, 43.2)	42.6 (40.0, 45.6)	< 0.01
*Remote pressure pain threshold*	13 (10, 17)	14 (10, 18)	0.4
*Remote pressure pain tolerance*	35 (29, 43)	59 (50, 75)	< 0.001
*Temporal summation*	0.62 (0.08, 1.23)	0.90 (0.27, 1.52)	0.1
*Conditioned pain modulation*	3 (− 1, 6)	5 (0, 12)	0.03

Median values (Interquartile range) or count (frequency) in the two groups derived from latent profile analysis. Univariate statistical tests performed: Wilcoxon rank-sum test or chi-squared test of independence. QST = Quantitative sensory pain testing

### Outcomes following spinal manipulation

#### Patient-reported outcomes

**Fig. 3:**
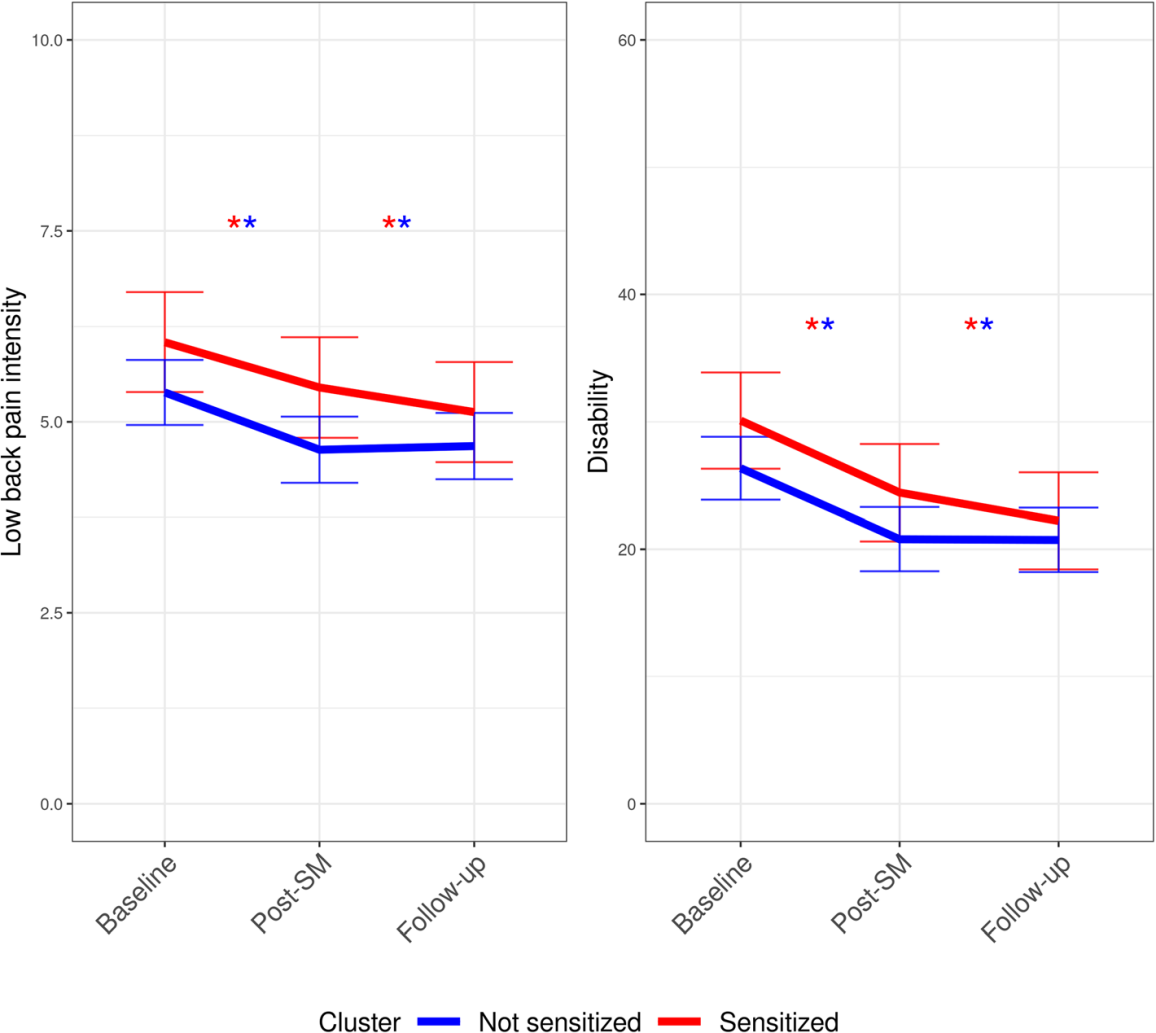
Within-cluster mean changes in low back pain intensity and disability for each latent profile derived cluster after spinal manipulation. Estimated marginal means (95% confidence intervals) from baseline to post-SM and follow up, respectively, in the two groups derived from latent profile analysis. Low back pain intensity is measured on a numerical pain rating scale [0–10]. Disability is measured using the Oswestry disability index [0–100], * = a within-cluster p-value < 0.05

Table 5 and Fig. 3 presents the outcomes from baseline to post-SM and follow-up following SM on low back pain intensity and disability. Although no between-cluster mean differences were found, both groups displayed statistically significant within-cluster mean changes, but not clinically important reductions in ODI and NRS.

**Table 5:** Within- and between-cluster changes in low back pain intensity and disability for each latent profile derived cluster after spinal manipulation

Outcomes		Baseline to Post-SM		Baseline to Follow up	
		Sensitized	Not sensitized	Sensitized	Not sensitized
*Low back pain intensity (NRS)*	Within-group changes	−0.59(− 1.14 to −0.04)*	−0.75(− 1.11 to −0.39)*	−0.92(− 1.46 to − 0.37)*	− 0.75(− 1.11 to − 0.39)*
	Between-group differences	− 0.16(− 0.82 to 0.5)		0.21(− 0.44 to 0.87)	
*Disability (ODI)*	Within-group changes	−5.67(− 8.79 to −2.54)*	−5.57(− 7.64 to − 3.49)*	−7.88(− 10.98 to − 4.77)*	− 5.57(− 7.64 to − 3.49)*
	Between-group differences	0.1(− 3.65 to 3.85)		2.26(− 1.48 to 5.99)	

Mean within-cluster changes (95% Confidence intervals) and mean between-cluster differences (95% confidence intervals) from baseline to post-SM and follow up, respectively, in the two groups derived from latent profile analysis. NRS = Numerical pain rating score [0–10], ODI = Oswestry disability index [0–100], * = *p*-value < 0.05

#### Quantitative sensory testing outcomes

Table 6 presents the within-cluster mean changes and between-cluster mean differences for the different QST parameters. For regional pressure pain threshold, both groups at both time points demonstrated an increase. However, at a much larger rate in the Sensitized group, resulting in a significant between-cluster difference post-SM. Remote pain tolerance also improved for the Sensitized group and regional heat pain threshold for the Not sensitized group. However, this only reached statistical significance at follow-up. In contrast, the temporal summation score increased (indicating more sensitization) for the Sensitized group at both time points – showing statistically significant between-cluster mean differences. All other parameters stayed consistent.

**Table 6:** Within and between changes in quantitative sensory tests for each latent profile derived cluster after spinal manipulation

Quantitative sensory testing		Baseline to Post-SM		Baseline to Follow up	
		Sensitized	Not sensitized	Sensitized	Not sensitized
*Regional pressure pain threshold*	Within-group changes	123 (72 to 174)*	52 (18 to 86)*	103 (52 to 155)*	52 (18 to 87)*
	Between-group differences	−71(− 132 to −9)*		− 51(− 113 to 11)	
*Regional heat pain threshold*	Within-group changes	0.1(−0.7 to 0.9)	0.2(− 0.3 to 0.7)	0.8(− 0.1 to 1.6)	0.6 (0 to 1.1)*
	Between-group differences	0.1(−0.9 to 1)		−0.2(− 1.1 to 0.8)	
*Remote pressure pain threshold*	Within-group changes	−1(− 4.1 to 2.1)	0.8(− 1.3 to 2.8)	2.3(− 0.8 to 5.3)	1.8(− 0.3 to 3.8)
	Between-group differences	1.8(− 2 to 5.5)		− 0.5(− 4.2 to 3.2)	
*Remote pressure pain tolerance*	Within-group changes	3.7(− 1 to 8.4)	− 0.1(− 3.1 to 3)	7.9 (3.3 to 12.5)*	3(−0.1 to 6.1)
	Between-group differences	−3.8(− 9.4 to 1.8)		− 4.9(− 10.4 to 0.7)	
*Temporal summation*	Within-group changes	0.6 (0.1 to 1)*	−0.3(− 0.6 to 0)	0.6 (0.2 to 1.1)*	− 0.2(− 0.5 to 0.1)
	Between-group differences	− 0.8(− 1.3 to − 0.3)*		−0.8(− 1.4 to − 0.3)*	
*Conditioned pain modulation*	Within-group changes	0(− 3.9 to 3.9)	0.3(−2.2 to 2.9)	−0.8(− 4.6 to 3.1)	0.6(− 2 to 3.1)
	Between-group differences	0.3(−4.3 to 5)		1.3(− 3.3 to 5.9)	

Mean within-cluster changes (95% Confidence intervals) and mean between-cluster differences (95% confidence intervals) from baseline to post-SM and follow up, respectively, in the two groups derived from latent profile analysis. PPT: Regional pressure pain threshold [kPa, 0–1000]. HPT: Regional heat pain threshold [Degrees celsius, 32–50], rPPT: Remote pressure pain threshold [kPa, 0–100], rPTT: Remote pressure pain tolerance threshold [kPa, 0–100], TS: Temporal summation [visual analog scale, 0–10], CPM: Conditioned pain modulation [visual analog scale, 0–10]. * = *p*-value < 0.05

#### Posthoc analysis

##### Does a shift in cluster classification imply clinical improvements

**Fig. 4:**
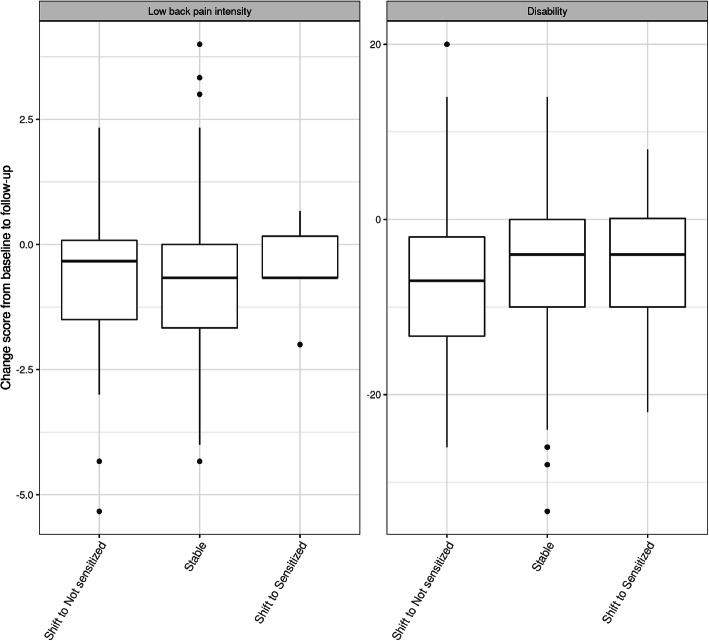
The association between clinical improvements and change in cluster classification following spinal manipulation. The y-axis presents the change scores in patient-reported outcomes between follow-up and baseline. A negative value indicates improvement. The x-axis presents the stability of the clusters from baseline to follow-up. Low back pain intensity is measured on a numerical pain rating scale [0–10]. Disability is measured using the Oswestry disability index [0–100]. Stable (*n* = 78), Shift to Not sensitized (*n* = 20), Shift to Sensitized (*n* = 7)

We completed a posthoc analysis of whether a change in cluster classification was associated with more considerable clinical improvements. Of the 105 clustered participants: 78 did not change cluster, 20 shifted from Sensitized to Not sensitized, and 7 from Not sensitized to Sensitized. We present a descriptive box plot in Fig. 4 of the changes observed in NRS and ODI from baseline to follow-up based on cluster change. Visual analysis does not indicate any differences; the medians all appear similar, and the variance does not differ greatly.

##### Segment randomization

**Fig. 5:**
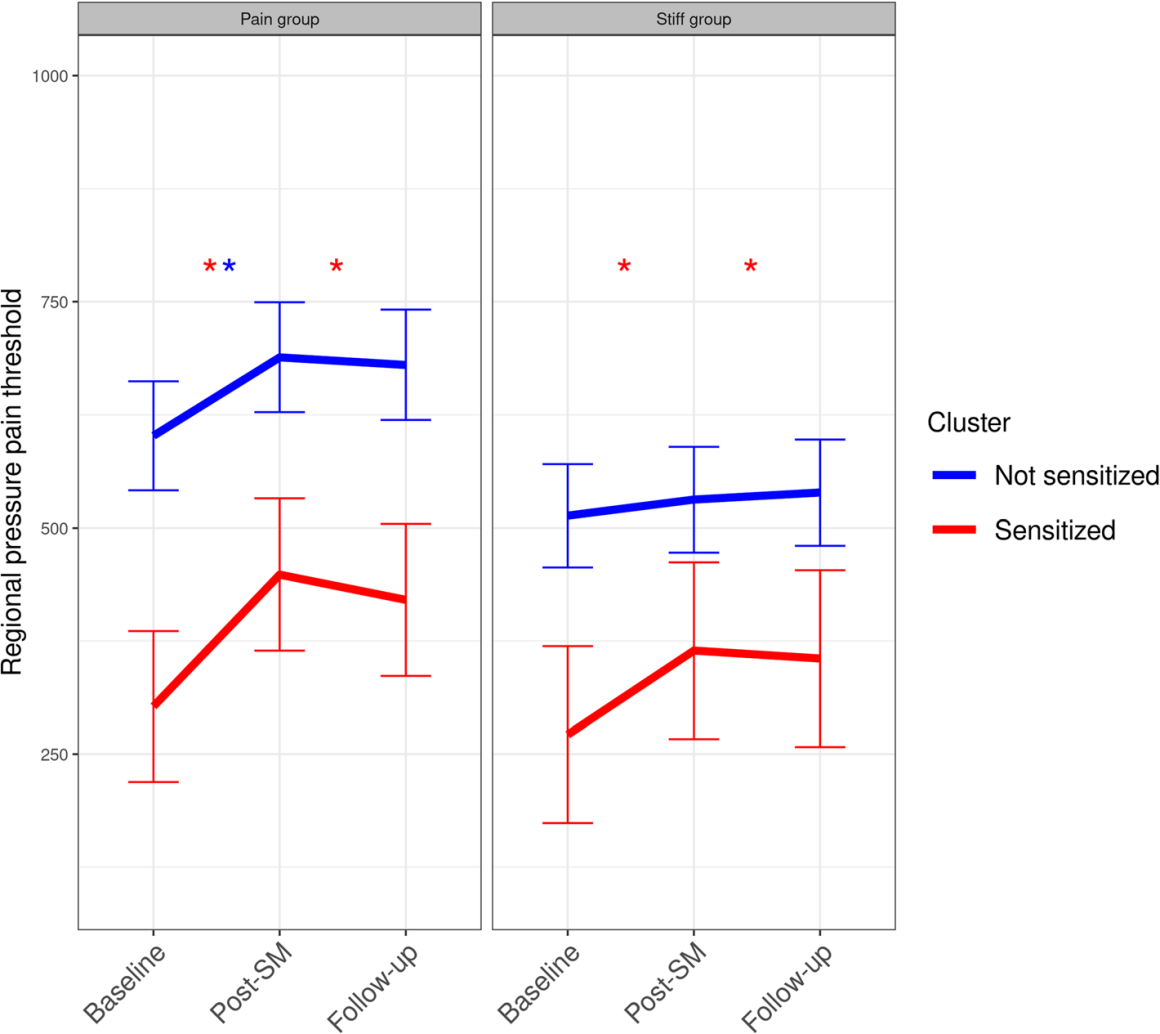
Within changes in regional pressure pain threshold [kPa, 0–100] for each latent profile derived cluster faceted by segment allocation after spinal manipulation. Estimated marginal means (95% confidence intervals) from baseline to post-SM and follow up, respectively, in the two groups derived from latent profile analysis for each allocated SM segment group. * = a within-cluster p-value < 0.05

A posthoc analysis (cluster:segment: SM allocation site) showed consistency with our prior findings [17]. We observed increases of regional pressure pain threshold in two instances i) the group receiving SM at a pain-sensitive segment independent of cluster classification and ii) the Sensitized group independent of allocation site. See Table 7 and Fig. 5.

**Table 7:** Within changes in regional pressure pain threshold [kPa, 0–100] for each latent profile derived cluster faceted by segment allocation after spinal manipulation

Cluster group	Treatment allocation	Baseline to Post-SM	Baseline to Follow-up
*Sensitized*	Pain segment group	146 (68:224)*	118 (40:196)*
	Stiff segment group	93 (3:183)*	84(−6:174)
*Not Sensitized*	Pain segment group	87 (31:143)*	78 (22:135)*
	Stiff segment group	18(−37:73)	26(− 30:81)

Within mean changes (95% confidence intervals) from baseline to post-SM and follow up, respectively, in the two groups derived from latent profile analysis for each allocated SM segment group

##### Temporal summation

We examined whether the reverse relation observed between temporal summation and sensitivity status was due to range-of-instrument constraints. We examined the distribution of the pressure pain intensity score from which temporal summation was calculated. Potentially, very low or very high pressure pain intensity scores could have resulted in ceiling or flooring effects, thus obfuscating any temporal summation effect. However, the Sensitized group scores were located at the center of the VAS-scale, and the pressure pain intensity ranged from a mean VAS-score of 3.75 (pulse 1 to 3) to 4.40 (pulse 8 to 10). Similarly, for the Not sensitized group, the range was 4.09 to 5.06. We also ran the latent profile analysis reversing the temporal summation score to the original score. Unsurprisingly, this did not result in any differences.

## Discussion

To our knowledge, this was the first study to investigate variations in pain sensitivity using a comprehensive QST battery in a large cohort of persistent LBP patients receiving SM. The latent profile analysis clustered two groups: a *Sensitized* and a *Not sensitized*. This division is not surprising as QST scores often correlate independent of the pain domain [42], and similar findings have been reported previously [43]. However, and in contrast to the prior findings [43], the clusters only showed minor differences in patient-reported measures, demographics, and psychological variables, none of which reached statistical significance.

The categorization into clusters was not associated with overall clinical improvement, which confirms previous publications concerning the predictive value of QST in LBP [7]. In general, we observed only minor clinical improvements, which could be due to an inefficient treatment paradigm for the select patient cohort under study. We have no data to support that assertion directly. However, we included participants from a patient population seen at a secondary care hospital Spine Center, for which referral criteria stipulate that relevant conservative treatment has already been undertaken in a primary care setting but proven unsuccessful.

Despite the limited clinical improvements, we found substantial regional pressure pain threshold increases, and while consistent with the literature [16], the Sensitized group’s changes were surprisingly large. Arguably, two parallel mechanisms can explain this increase in regional pressure pain threshold: (I) A change following SM on pain sensitivity in those with generalized high pain sensitivity, and (II) a segmental reflex effect of SM when directed at a sensitized segment (independent of generalized high pain sensitivity). While mechanism II appears to be a causal effect due to the application site’s randomization, we cannot state the same about mechanism I. This could solely be due to regression towards the mean. However, the observation that the Sensitized group scored significantly lower in several pain measures at baseline, whereas only regional pressure pain threshold changed in such an extensive and systematic manner following SM, speaks against regression towards the mean.

Furthermore, shifting from a Sensitized to a Not sensitized cluster was not associated with an increased clinical reduction, compared to not shifting or shifting to a Sensitized cluster. However, using the same data, we have previously demonstrated that being classified as a clinical responder was associated with regional pressure pain threshold changes but not regional heat pain threshold [44]. Thus, the relationship between clinical improvement and changes in regional pain sensitivity is not straight forward.

### Regional mechanical pain sensitivity

Arguably, this provides new evidence that generalized sensitization appears to be a modifier for changes in regional pressure pain threshold following SM. However, if we attribute such an effect to SM, it appears strictly limited to regional mechanical pain sensitivity rather than a systemic or remote effect. The minor but statistically significant increase in regional heat pain threshold is simply a testament to the larger group size in the Not sensitized group. This decrease was only slightly lower than for the Sensitized group (Δ 0.2 degrees difference). This supports the literature, namely that thermal pain threshold is not affected by SM [14, 45].

We did find a heterotopic effect of SM concerning remote pressure pain threshold, but this was only observed at follow-up two weeks later and not in the immediate post-SM period. It seems unlikely that this should reflect a delayed remote effect of SM when we did not observe any differences in patient-reported pain and disability.

We observed a systematic inverse difference in temporal summation for both groups, indicating higher degrees of pain sensitization in the Sensitized group and vice versa in the not sensitive group. While not due to any constraint introduced by the VAS-scale, arguably, the remote pressure pain tolerance thresholds were so low in the Sensitized group that the repeated stimuli did not provide sufficient input to facilitate a wind-up phenomenon. Thereby not inducing an observable temporal summation effect [46]. This analysis and a recent randomized trial comparing SM to sham treatment found no effect on temporal summation [47]. Thus, SM appears not to affect temporal summation.

To the authors’ knowledge, this is the first study to examine within-cluster changes of conditioned pain modulation following SM of the lower back. An attenuated conditioned pain modulation response has been suggested to be a central component in the development of persistent pain [48], and our latent profile analysis supports this. However, as administered in this study setting, SM did not affect the conditioned pain modulation response, but as with temporal summation, we saw limited scores for conditioned pain modulation. The low remote pressure pain tolerance resulted in a very low conditioning stimulus (70% of tolerance) and, thus, potentially did not sufficiently activate the central inhibition needed to modulate the subsequent pain stimuli.

To summarize, the limited changes in temporal summation and conditioned pain modulation following SM could be a limitation of the CCA protocol. Potentially, the protocol did not adequately facilitate these phenomena.

### Methodological considerations

A strength of the current study was the large number of participants with persistent LBP. We found the inclusion of persistent LBP patients appropriate, as this study aimed to investigate the mechanism related to changes in pain sensitivity. Perturbed pain modulation is likely more prevalent in our cohort of persistent pain patients seen at a hospital than primary care patients. Nevertheless, it is unclear whether to expect higher or lower SM effects in a population with more pronounced perturbation of pain modulation. Conversely, the study cohort was drawn from a patient population that had failed to respond to conservative treatment and was probably less likely to respond to the SM intervention than a primary care patient group.

We choose to use the absolute values for the latent profile analysis in our regression models. The participants’ probability of belonging to that particular group was high, and four of the six QST parameters differed statistically significantly between the groups, despite substantial variation for each measure. Furthermore, the clustering was not indirectly affected by demographic or physiological variables with possible affiliations to the QST measures.

We used a large QST battery that covered multiple techniques, stimuli, and pain domains, all tested by the same rater. By contrast, many, if not most studies investigating changes in pain sensitivity, apply only a single QST [13–16]. The SM’s standardization was arguably a weakness of the intervention due to the simplicity and dissimilarity with clinical practice, i.e., poor external validity. However, it did optimize the underlying hypothesis of investigating the SM’s mechanism, increasing the reproducibility. As stated previously, this was not a placebo-controlled trial, so we cannot state whether the observed changes were causally linked.

## Conclusion

Distinct subgroups (Sensitized and Not sensitized) were identified from the latent profile analysis. However, the clusters were not associated with changes in clinical outcomes, regional thermal pain thresholds, or remote static and dynamic quantitative sensory tests.

SM’s cluster affiliation and application site were related to the observed change in regional mechanical pain sensitivity by two parallel mechanisms. I) Changes following SM specific to participants with generalized high pain sensitivity, and II) local effects of SM application when applied at a sensitized segment independent of general high pain sensitivity. Thus, the mechanism that involves clinical improvement appears to be separate from that which produces changes in pain sensitivity.

These results are only specific to this cohort of persistent LBP patients sampled from a hospital and should not be generalized to a primary care setting.

## Supplementary Information

**Additional file 1 Supplementary material 1**. A sketch of the 3D-printed double headed probe.

**Additional file 2 Supplementary material 2**. Correlation matrix. Legend: The correlation between the six quantitative sensory test parameters. rPPT = regional pressure pain threshold, rHPT = regional heat pain threshold, wPPT = remote pressure pain threshold, wPTT = remote pressure pain tolerance threshold, TS = Temporal summation, CPM = conditioned pain modulation.

## Data Availability

Data is available upon reasonable request. Please contact casper.nim@rsyd.dk.

## Abbreviations

QST: Quantitative sensory test
LBP: Low back pain
SM: Spinal manipulation
NRS: Numerical pain rating score
ODI: Oswestry disability index
PPT: Regional pressure pain threshold
HPT: Regional heat pain threshold
CCA: Computer-controlled cuff algometry
rPPT: Remote pressure pain threshold
rPTT: Remote pressure pain tolerance threshold
TS: Temporal summation
CPM: Conditioned pain modulation
VAS: Electronic visual analog scale
BIC: Bayesian Information Criterion
