# Supplementary figures and images for: Spinal manipulation and modulation of pain sensitivity in persistent low back pain: a secondary cluster analysis of a randomized trial
Source: Chiropr Man Therap. 2021 Feb 24;29:10. doi: 10.1186/s12998-021-00367-4 (PMC7903787; doi:10.1186/s12998-021-00367-4)

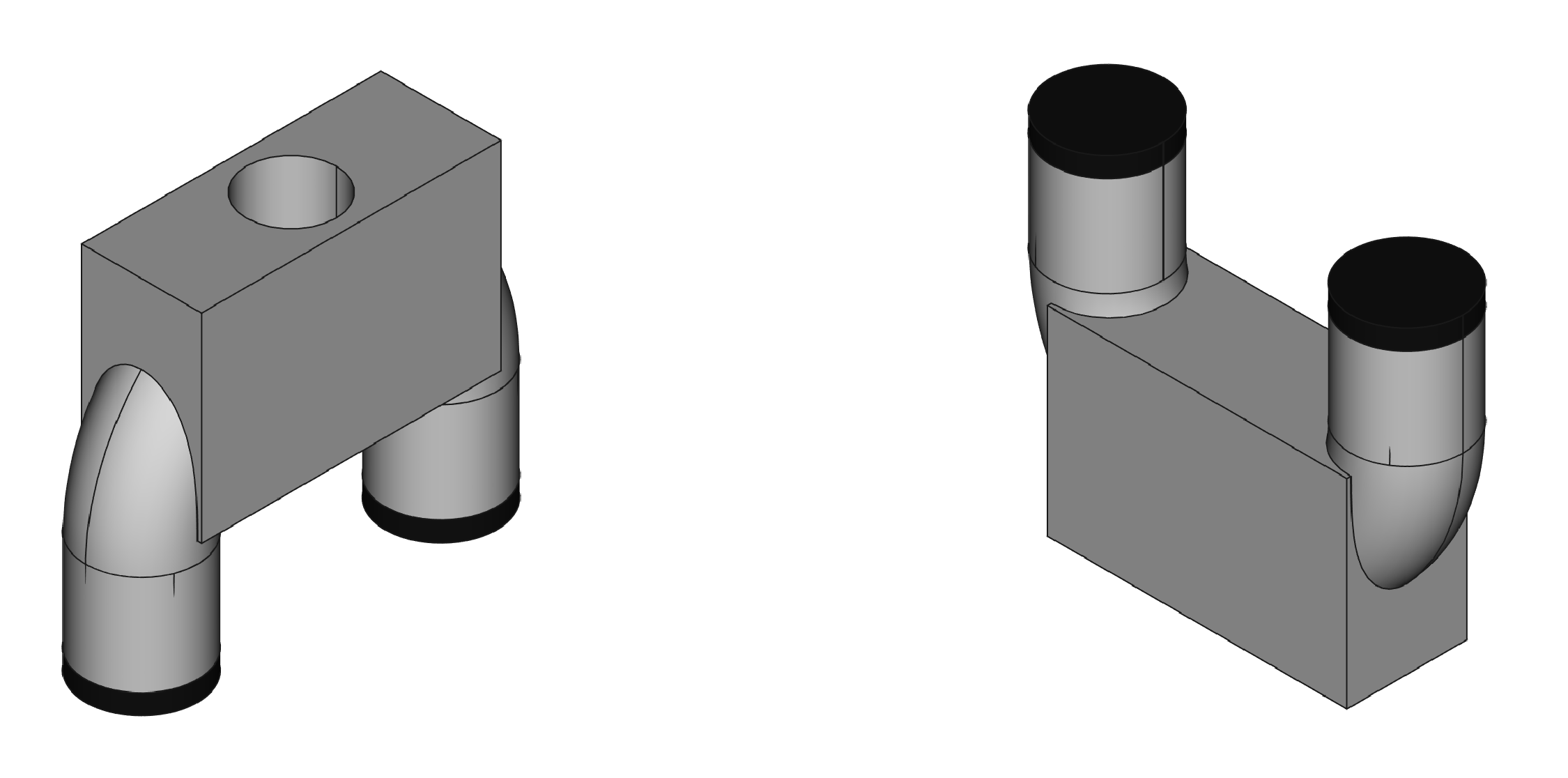

Supplement: Supplementary file 1 — Additional file 1 Supplementary material 1. A sketch of the 3D-printed double headed probe. [file 12998_2021_367_MOESM1_ESM.png]

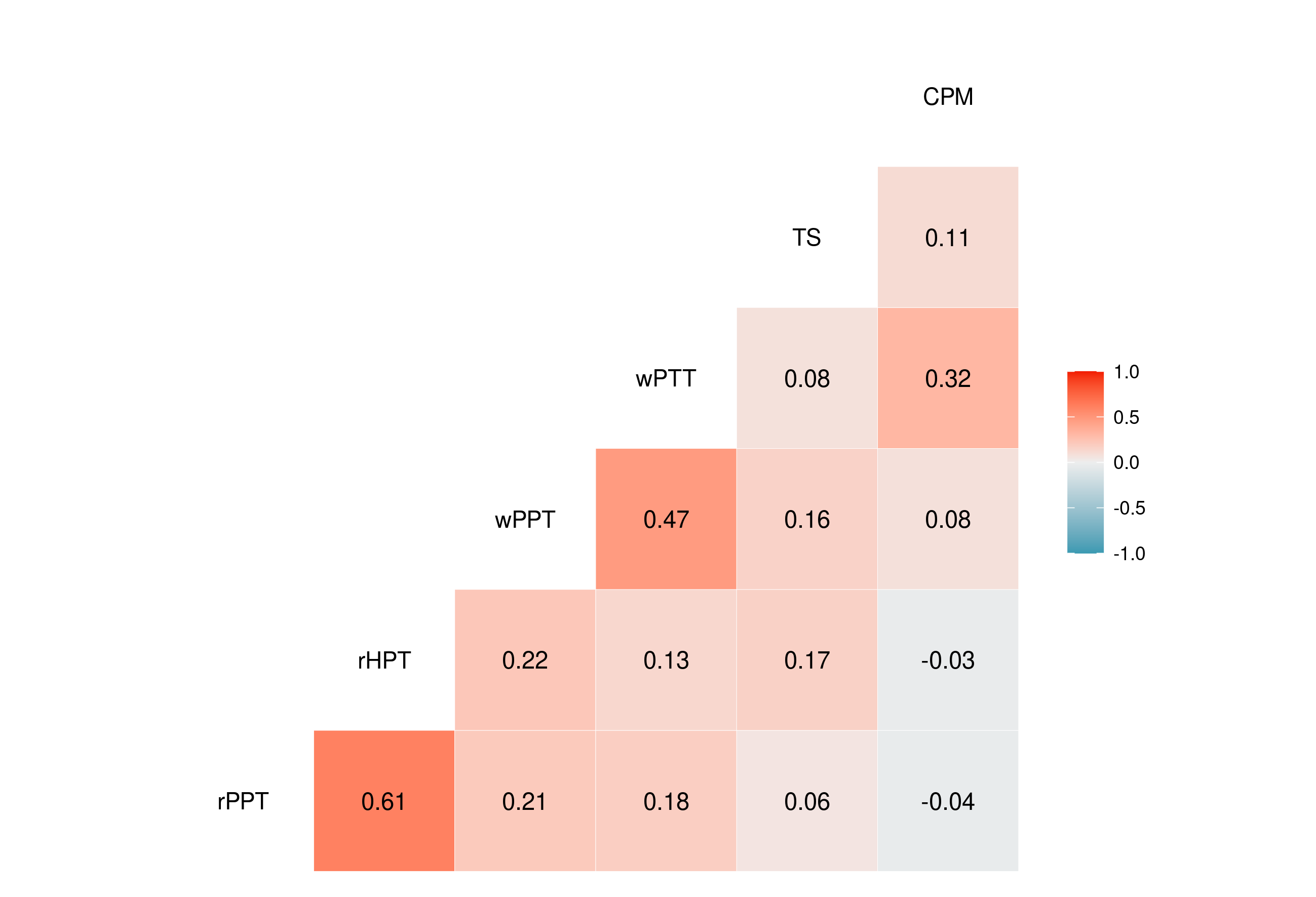

Supplement: Supplementary file 2 — Additional file 2 Supplementary material 2. Correlation matrix. Legend: The correlation between the six quantitative sensory test parameters. rPPT = regional pressure pain threshold, rHPT = regional heat pain threshold, wPPT = remote pressure pain threshold, wPTT = remote pressure pain tolerance threshold, TS = Temporal summation, CPM = conditioned pain modulation. [file 12998_2021_367_MOESM2_ESM.png]
